# Rapid magneto-enzyme-linked immunosorbent assay for ultrasensitive protein detection

**DOI:** 10.1016/j.aca.2022.340246

**Published:** 2022-09-08

**Authors:** Kavya L. Singampalli, Jiran Li, Peter B. Lillehoj

**Affiliations:** a Department of Bioengineering, Rice University, 6500 Main St. Houston, TX, 77030, USA; b Department of Mechanical Engineering, Rice University, 6100 Main St. Houston, TX, 77005, USA; c Medical Scientist Training Program, Baylor College of Medicine, One Baylor Plaza Houston, TX, 77030, USA

**Keywords:** ELISA, Magnetic nanoparticles, Immunoassay, *Pf*HRP2, SARS-CoV-2

## Abstract

Protein-based diagnostics are the standard of care for screening and diagnosing a broad range of diseases and medical conditions. The current gold standard method for quantifying proteins in clinical specimens is the enzyme-linked immunosorbent assay (ELISA), which offers high analytical sensitivity, can process many samples at once, and is widely available in many diagnostic laboratories worldwide. However, running an ELISA is cumbersome, requiring multiple liquid handling and washing steps, and time-intensive (∼2 − 4 h per test). Here, we demonstrate a unique magneto-ELISA that utilizes dually labeled magnetic nanoparticles (DMPs) coated with horseradish peroxidase (HRP) and an HRP-conjugated detection antibody, enabling rapid immunomagnetic enrichment and signal amplification. For proof of concept, this assay was used to detect *Plasmodium falciparum* histidine-rich protein 2 (*Pf*HRP2), a malaria parasite biomarker, which exhibited a lower limit of detection of 2 pg mL^−1^ (33 fM) in human serum. Measurements of *Pf*HRP2 in clinical blood samples from individuals with and without *P. falciparum* infection revealed that this magneto-ELISA offers a superior diagnostic accuracy compared to a commercial *Pf*HRP2 ELISA kit. We also demonstrate the versatility of this platform by adapting it for the detection of SARS-CoV-2 nucleocapsid protein, which could be detected at concentrations as low as 8 pg mL^−1^ (174 fM) in human serum. In addition to its high analytical performance, this assay can be completed in 30 min, requires no specialized equipment, and is compatible with standard microplate readers and ELISA protocols, allowing it to integrate readily into current clinical practice.

## Highlights

• A unique magneto-ELISA was developed for rapid, ultrasensitive protein detection.
• This assay exhibits a lower limit of detection of 2 pg mL^−1^ (33 fM) in human serum.
• Each measurement is completed in 30 min, making it at least 4× faster than ELISA.
• Measurements of clinical samples reveal superior accuracy to a commercial ELISA kit.
• This assay is compatible with standard microplate readers and ELISA techniques.

## Graphical abstract

**Figure:**
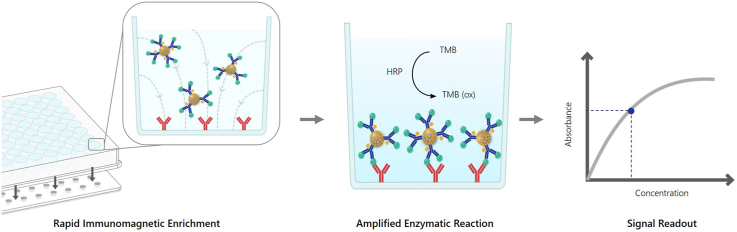

## 1 Introduction

The detection and quantification of protein biomarkers are used for a broad range of clinical applications, including disease diagnosis and screening, assessing therapeutic response, and monitoring disease progression [[1], [2], [3], [4]]. The current gold standard technique for quantitative protein detection in clinical specimens is the enzyme-linked immunosorbent assay (ELISA). ELISA offers the benefits of high sensitivity measurements, with most commercial ELISA kits claiming a lower limit of detection (LOD) in the 100's of pg mL^−1^ range [5], and high specificity, resulting from its use of antigen-antibody pairs. In addition, ELISA can process many samples at once due to its format in a 96-well microplate, making it useful for large-scale testing and screening. Due to these advantages, ELISA is recommended by the World Health Organization as an essential diagnostic modality [6], and as such is widely available in many diagnostic laboratories worldwide. While ELISA offers many benefits as a diagnostic technique, one of its main drawbacks is that it involves multiple incubation and wash steps, making the overall procedure laborious and time-consuming (∼2 − 4 h per test). The extended time and labor required for conventional ELISA hinder its use for applications requiring short turnaround times, such as on-site diagnostic testing and high-throughput screening.

To reduce the time and complexity associated with ELISA, various techniques have been developed to enhance the kinetics of antigen-antibody binding, amplify the detection signal produced by the enzymatic reporter or simplify the testing protocol. Dixit et al. employed covalent immobilization of the capture antibody in the ELISA microwell which, when compared to passive adsorption, resulted in a ∼10-fold improvement in the LOD for the detection of human fetuin A [7]. Additionally, nanoparticles have been used as carriers for detection antibodies and/or reporter molecules, which can amplify the detection signal due to their large surface area and presence of multiple active binding sites, allowing them to carry a large number of detection molecules [8]. Ambrosi et al. demonstrated that the use of gold nanoparticles coated with detection antibody (dAb)-reporter conjugates resulted in 2-fold higher sensitivity for detecting cancer antigen 15-3 with a significantly reduced enzymatic reaction time compared with the use of free dAb [9]. Magnetic nanoparticles offer the further advantage of localization, as they can be rapidly concentrated using an external magnetic field. This facilitates the transport of biomolecules in the sample, which reduces the time needed for immunocomplex formation and enables rapid, simple separation/concentration of biological species within an immunoassay. This technique has been demonstrated for the rapid transfer of magnetic bead-conjugated immune complexes between reagents and wash buffers, enabling the detection of anti-SARS-CoV-2 antibodies within 15 min [10]. In addition to reducing the assay time, the use of magnetic nanoparticles has also been shown to enhance the colorimetric signal of ELISA by transferring analyte-magnetic bead complexes from a large sample volume, thus concentrating a dilute analyte within a small volume at the microwell surface [11]. These techniques have leveraged the ability of an external magnetic field to rapidly isolate magnetic bead complexes, resulting in lower LODs and faster detection times.

An alternative strategy to enhance the analytical sensitivity of ELISA has been to modify the enzyme reporter. In conventional ELISA protocols, horseradish peroxidase (HRP) is used as the enzyme reporter and undergoes an oxidation reaction in the presence of 3,3′,5,5′-Tetramethylbenzidine (TMB) substrate, resulting in a colorimetric signal that is proportional to the concentration of the target analyte on the microwell surface. Therefore, increasing the amount of HRP that is attached to the target analyte can amplify the detection signal, allowing for lower protein concentrations to be detected. This was demonstrated by Wang et al. who functionalized nanoparticles with biotin and poly(amidoamine) to bind additional HRP molecules, which increased the detection signal by 10-fold [12]. Similarly, de la Serna et al. reported the use of poly-HRP, a polymeric unit of HRP that produces a color change equivalent to multiple molecules of HRP, in conjunction with magnetic nanobeads for the detection of *Plasmodium falciparum* lactate dehydrogenase (*Pf*LDH) in lysed whole blood. This modified ELISA exhibited a LOD of 0.11 ng mL^−1^ and assay time of 1 h [13], indicating that increasing the concentration of enzymatic reporter can improve the sensitivity and reduce the detection time.

The approaches described above have been successful in either enhancing the analytical sensitivity of ELISA, reducing the assay time, or simplifying the testing protocol; however, there is currently no ELISA technique that can offer ultrasensitive (single pg mL^−1^) protein detection in clinical samples in ≤30 min. While there are other types of rapid immunoassays (e.g., chemiluminescence, fluorescence, electrochemical [14,15]) that can detect proteins with high sensitivity, they require specialized instrumentation, involve complicated protocols, or are not suitable for testing large numbers of samples at once. To overcome these limitations, we have developed a rapid (30 min) magneto-ELISA for ultrasensitive protein measurements in purified and whole blood samples, which does not require specialized instrumentation and is compatible with standard microplate readers and ELISA protocols. This novel assay utilizes dually labeled magnetic nanoparticles (DMPs) that are coated with HRP and an HRP-conjugated dAb. Each DMP contains multiple dAb molecules, increasing the number of binding sites for the target antigen, as well as multiple HRP molecules, resulting in a substantial enzymatic reaction and amplified detection signal. Additionally, this assay utilizes a rapid and simple immunomagnetic enrichment technique to transport antigen-DMP immunocomplexes to the capture antibody (cAb)-immobilized microwell surface, which enhances the kinetics of sandwich immunocomplex formation. We show that this magneto-ELISA can be readily adapted to detect other protein biomarkers in different types of clinical samples, including human plasma, serum, and whole blood, while maintaining high analytical sensitivity, showcasing its versatility as a diagnostic assay.

## 2 Materials and methods

### 2.1 Biochemicals and reagents

10 mM Tris hydrochloride buffer (pH = 8.0) and 0.01 M phosphate buffered saline (pH = 7.4) were purchased from Bioworld Inc. and Sigma-Aldrich, respectively. 0.1 M 2-(N-morpholino) ethanesulfonic acid (MES, pH = 4.7) buffer was purchased from Thermo Fisher Scientific and diluted to 25 mM using deionized water. Washing buffer was prepared by diluting Tween-20 (Sigma-Aldrich) in PBS to produce a 0.05% (w/v) Tween-20 solution. N-(3-dimethylaminopropyl)-N-ethylcarbodiimide (EDC) was purchased from Thermo Fisher Scientific. N-hydroxysuccinimide (NHS) and horseradish peroxidase (HRP) were purchased from Sigma-Aldrich. Enhanced K-Blue TMB substrate was purchased from Neogen Inc. Matrix Guard Diluent, StabilBlock Immunoassay Stabilizer, StabilCoat Plus Immunoassay Stabilizer and Staiblzyme HRP Conjugate Stabilizer were purchased from Surmodics Inc. Human sera was purchased from Sigma-Aldrich and human plasma and whole blood were purchased from BioIVT. Blood samples from donors with *P. falciparum* infection obtained in Uganda under IRB/EC approval for general research use were purchased from Discovery Life Sciences. All human samples were de-identified of identifying information.

### 2.2 Fabrication of the magnetic stage

The magnetic stage consists of an array of 96 1/8-inch diameter neodymium magnets (McMaster Carr) with centers positioned 9 mm apart in a laser-cut poly methyl methacrylate (PMMA) base that fits a standard 96-well microplate (Fig. S1). The base consists of 3 layers of PMMA joined together using double-sided tape to provide a height of 3 mm, which ensures that each magnet is in contact with the bottom of the microplate and centered under each well.

### 2.3 Preparation of microplates

Anti-*Plasmodium falciparum* histidine-rich protein 2 (*Pf*HRP2) IgG (Fitzgerald Industries International) or anti-SARS-CoV-2 N protein IgG (Arigo Biolaboratories) was diluted in Tris-HCl buffer to a concentration of 10 μg mL^−1^. 45 μL of the diluted antibody solution was added to each well of a high-bind polystyrene 96-well plate (Corning), incubated at 4 °C for 16 h to allow for passive adsorption of the antibody to the microplate, then washed with 0.05% Tween-20. 300 μL of StabilBlock Immunoassay Stabilizer was added to each well, incubated for 1 h, and removed by tapping the microplate upside down. Prepared microplates were dried overnight at 4 °C and used immediately or vacuum sealed and stored at 4 °C for up to one month.

### 2.4 Preparation of dually labeled magnetic nanoparticles (DMPs)

DMPs were prepared by binding HRP and anti-*Pf*HRP2 IgG-HRP conjugates (ICL Inc.) or anti-SARS-CoV-2 nucleocapsid (N) protein IgG-HRP conjugates (GeneTex) to 200 nm carboxylated magnetic nanobeads (Ademtech) using carbodiimide chemistry, as previously described [16]. Briefly, 1 mg of magnetic beads was washed in 25 mM MES buffer, followed by shaking at 500 rpm with 200 μL of EDC/NHS (10 mg mL^−1^ in 25 mM MES) for 50 min. The beads were then mixed with 50 μL of HRP-conjugated detection antibody (50 μg mL^−1^) and 50 μL of HRP (3mg mL^−1^) in 25 mM MES (1:200 IgG:HRP molar ratio). The bead-protein mixture was shaken overnight (15 h), washed six times with PBS, incubated twice with StabilCoat Plus Immunoassay Stabilizer for 45 min each, and stored in 400 μL of StabilZyme HRP Conjugate Stabilizer. DMPs were used immediately or stored at 4 °C for up to 2 weeks.

### 2.5 ELISA measurements

*Pf*HRP2 (CTK Biotech), *P. falciparum* lactate dehydrogenase (*Pf*LDH, CTK Biotech), *Plasmodium* aldolase (CTK Biotech) or SARS-CoV-2 N protein (Advaite, Inc) was spiked in human sera, plasma or whole blood diluted 10× in MatrixGuard diluent (Surmodics, Inc) to generate simulated samples for assay optimization and testing. The simulated sample was first combined with DMPs at a 1:40 volume ratio and 85 μL of the sample-DMP mixture was immediately added to each well. The microplate was then incubated on an orbital shaker for 14 min at 300 rpm. The micropalte was placed on the magnetic stage for 1 min for magnetic concentration and then incubated without agitation at room temperature for 5 min, followed by a wash step. Washing was performed by dispensing 300 μL of 0.05% Tween-20 solution into each well and manually shaking the micrplate for 10 s. The solution was removed by flipping the microplate upside down and gently tapping on the bottom to remove residual liquid. This process was repeated six times, constituting a wash step. After washing, 100 μL of TMB substrate was added to each well and the microplate was incubated on an orbital shaker for 10 min at 150 rpm. 50 μL of 2 N H_2_SO_4_ was added to each well to stop the HRP-TMB reaction. The colorimetric signal was read using a BioTek Epoch microplate spectrophotometer at a wavelength of 450 nm. Measurements of deidentified clinical blood samples from malaria-positive and malaria RDT-negative individuals were performed using the same protocol as the simulated samples. Quantimal Ultrasensitive *Pf*HRP2 ELISA kits were purchased from Cellabs Inc. and run according to the manufacturer's protocol. The cut-off value for discriminating between positive and negative cases was defined as 0.1 + the average absorbance of the negative samples for the Cellabs kit (per manufacturer's instructions) and as the average absorbance (OD 450 nm) of the negative samples + 3 × standard deviation for the magneto-ELISA [17].

### 2.6 Statistical analysis

Statistical analysis was conducted using an unpaired Student's t-test between different testing parameters and a Spearman's rank correlation coefficient for comparison to standard ELISA techniques. Data analysis was conducted using GraphPad Prism 9.

## 3 Results and discussion

### 3.1 Principle of the magneto-ELISA

**Fig. 1:**
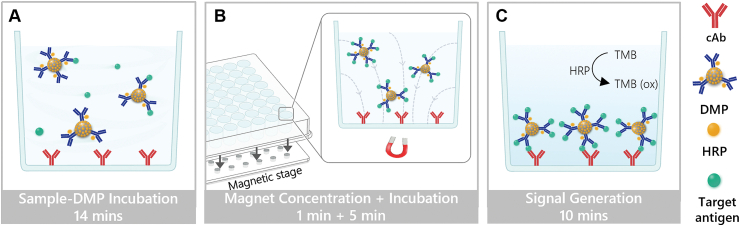
**Schematic of the magneto-ELISA testing protocol.** (A) Incubation of the sample with DMPs. (B) Magnetic concentration of antigen-DMP immunocomplexes on cAb-immobilized wells. (C) Generation of the colorimetric signal due to the HRP-catalyzed oxidation of TMB.

This assay is based on a conventional sandwich ELISA format where an antibody pair and enzyme reporter are used to detect a target antigen. Similar to a conventional sandwich ELISA, the cAb is immobilized on the bottom of the microplate well. However, our assay differs in its utilization of DMPs that are coated with HRP-conjugated dAb and free HRP, which allows for rapid immunomagnetic enrichment and enhanced signal amplification. To initiate the measurement, DMPs are added to the sample and the sample-DMP mixture is incubated in the wells (with agitation) for 14 min. If the sample contains the target antigen, it binds to the DMP and forms an antigen-DMP immunocomplex (Fig. 1A). The microplate is then placed on the magnetic stage, which generates a localized magnetic field under each well and causes the antigen-DMP immunocomplexes to rapidly migrate to the bottom of the well where they subsequently bind to the surface-immobilized cAb (Fig. 1B). In the presence of TMB substrate, the HRP-coated DMPs catalyze the oxidation of TMB, generating a colorimetric signal that is proportional to the concentration of target antigen attached to the cAb-immobilized well (Fig. 1C). If the sample does not contain the target antigen, the DMPs are washed away from the well and a negligible colorimetric signal is generated upon application of the TMB substrate. With the exception of the magnetic stage, which has an estimated cost of ∼USD $9 (Table S1 in Supplementary materials), this magneto-ELISA requires no additional parts or specialized instrumentation and is compatible with standard microplate readers.

**Fig. 2:**
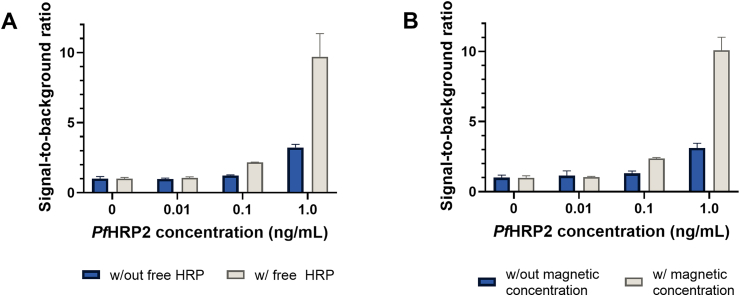
**Enhancement of ELISA with DMPs and magnetic concentration.** (A) Signal-to-background ratios generated from *Pf*HRP2-spiked human serum samples using magnetic particles labeled with HRP-conjugated dAb and free HRP (light bars) or particles labeled with HRP-conjugated dAb only (dark bars). Each bar represents the mean ± SD of three measurements. (B) Signal-to-background ratios generated from *Pf*HRP2-spiked human serum samples with 1 min of magnetic concentration (light bars) or with 30 min incubation without magnetic concentration (dark bars). Each bar represents the mean ± SD of four measurements.

The use of DMPs in this magneto-ELISA offers two major advantages over conventional ELISA. First, enhanced signal amplification is achieved with reduced incubation times because the DMPs are coated with HRP-conjugated dAb and free HRP. Since the colorimetric signal is generated from the reaction between HRP and the TMB substrate, the large amount of HRP on each DMP enhances the enzymatic reaction for a single surface-immobilized immunocomplex, resulting in a more substantial colorimetric signal. The improvement in the colorimetric signal was evaluated by using magnetic particles coated with HRP-conjugated dAb and free HRP or magnetic particles coated with HRP-conjugated dAb only for measurements of *Pf*HRP2 spiked in human sera. As shown in Fig. 2A, the signal-to-background ratios (SBRs) generated using magnetic particles coated with HRP-conjugated dAb and free HRP were up to 3-fold larger compared with those generated from the magnetic particles that contained HRP-conjugated dAb only. This result shows that immobilizing both free HRP and HRP-conjugated dAb on DMPs results in enhanced signal amplification without increasing the background signal, thereby improving the analytical sensitivity of the assay.

The other major advantage of this magneto-ELISA is that the immunomagnetic enrichment process accelerates the transport of antigen-DMP immunocomplexes to the bottom of the cAb-immobilized well, which enhances the immunoreaction kinetics, thereby increasing the likelihood of sandwich immunocomplex formation. The enhancement in immunocomplex formation due to magnetic concentration was studied by performing measurements of *Pf*HRP2-spiked human sera using the magneto-ELISA with 1 min of magnetic concentration or with 30 min of incubation without magnetic concentration. As shown in Fig. 2B, SBRs generated with magnetic concentration were up to 3-fold larger compared with those generated without magnetic concentration with a negligible change in the background signal. Therefore, the use of magnetic concentration further enhances the detection signal of the assay, while significantly reducing the incubation time to 30 min (compared to ∼2 − 4 h for conventional ELISA).

### 3.2 Optimization of assay parameters

**Fig. 3:**
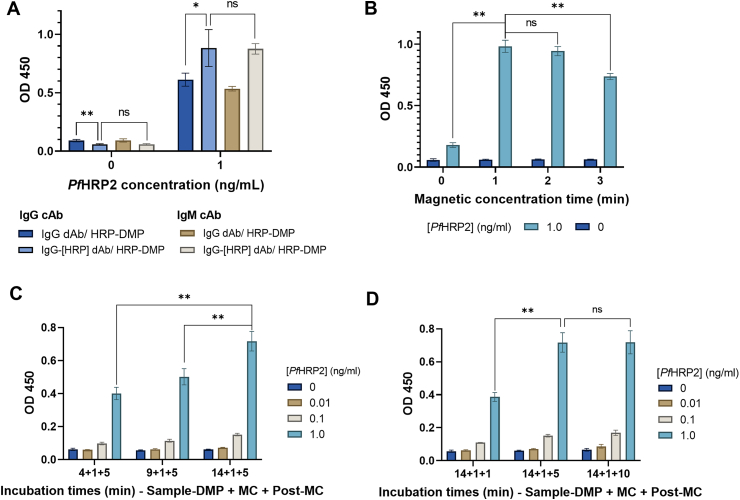
**Optimization of assay parameters.** (A) Absorbance values generated from the magneto-ELISA using human serum spiked with 1 ng mL^−1^ or 0 ng mL^−1^ of *Pf*HRP2 using different antibody pairs. (B) Absorbance values generated from *Pf*HRP2-spiked human serum with varying magnetic concentration (MC) durations, 14 min of sample-DMP incubation, and 5 min of post-MC incubation. (C) Absorbance values generated from *Pf*HRP2-spiked human serum with varying sample-DMP incubation durations, 1 min of MC, and 5 min of post-MC incubation. (D) Absorbance values generated from *Pf*HRP2-spiked human serum with varying durations of post-MC incubation, 14 min of sample-DMP incubation, and 1 min of MC. Each bar represents the mean ± SD of four measurements. * indicates p < 0.05, ** indicates p < 0.01.

Several assay parameters were optimized to maximize the SBR and minimize the variability in the detection signal. All assay optimization experiments were carried out using *Pf*HRP2 as the target analyte. The affinity of *Pf*HRP2 to the capture and detection antibodies was studied by performing measurements of human sera spiked with 1 ng mL^−1^ or 0 ng mL^−1^ of *Pf*HRP2 using different anti-*Pf*HRP2 antibody pairs. Both anti-*Pf*HRP2 IgG and IgM produced similar absorbance values when used as the cAb. However, the use of an HRP-conjugated anti-*Pf*HRP2 IgG dAb resulted in an ∼1.4-fold increase in the absorbance for the positive control sample (1 ng mL^−1^) and a reduction in absorbance for the negative control sample (0 ng mL^−1^) compared with those generated using anti-*Pf*HRP2 IgG dAb (Fig. 3A). The higher absorbance values generated by the HRP-conjugated anti-*Pf*HRP2 IgG are due to the additional HRP molecules bound to each DMP, thus increasing the enzymatic reaction and colorimetric signal produced by each surface-immobilized immunocomplex. Based on these results, anti-*Pf*HRP2 IgG was selected as the cAb and HRP-conjugated anti-*Pf*HRP2 IgG was selected as the dAb for the *Pf*HRP2 assay.

The durations of sample-DMP incubation, magnetic concentration, and post-magnetic concentration incubation were optimized to maximize the analytical sensitivity while reducing the assay time. First, the magnetic concentration duration was studied by performing measurements of *Pf*HRP2 spiked in human serum using varying magnetic concentration durations, which revealed that 1 − 2 min generated the highest absorbance values for all *Pf*HRP2 concentrations (Fig. 3B). We observed that magnetic concentration durations >2 min resulted in lower absorbance values since longer concentration times can cause an excessive amount of DMPs to be concentrated in a small area on the bottom of the well, which can hinder binding with the surface-immobilized cAb. To minimize the assay time, 1 min was selected as the optimal magnetic concentration duration. Sample-DMP and post-magnetic incubation times were then optimized to allow for completion of the assay within 30 min. Experiments to optimize the sample-DMP incubation duration were carried out using 4, 9, and 14 min of sample-DMP incubation with a 5 min post-magnetic concentration incubation time. As expected, longer sample-DMP incubation durations generated larger absorbance values since a longer incubation time allows for more antigen-antibody interactions and more antigen-DMP immunocomplex formation (Fig. 3C). Using 14 min as the sample-DMP incubation duration, experiments were performed to optimize the post-magnet concentration incubation duration using 1, 5, and 10 min. The absorbance values generated with 5 and 10 min of post-magnetic concentration incubation were similar for all *Pf*HRP2 concentrations and significantly larger than the absorbance values generated using 1 min of post-magnetic concentration incubation (Fig. 3D). Therefore, to minimize assay time, 5 min was selected as the optimal post-magnetic concentration incubation duration.

We further optimized the DMPs and incubation conditions to maximize the signal generated by the assay. The amount of DMPs added to the sample was optimized by performing measurements of human serum spiked with *Pf*HRP2 using varying sample-to-DMP solution volume ratios, as shown in Fig. S2A. The 40:1 and 20:1 sample-to-DMP volume ratios showed no significant difference, but both produced significantly higher absorbance values than those generated using the 80:1 sample-to-DMP volume ratio at all *Pf*HRP2 concentrations, indicating that the 40:1 ratio offers a sufficient amount of DMPs for immunocomplex formation for up to 1 ng mL^−1^ of target, while minimizing the consumption of magnetic particles and biochemicals. Additionally, the influence of the magnetic particle size on immunomagnetic enrichment performance was investigated by testing *Pf*HRP2-spiked serum samples using DMPs with diameters of 100 nm, 200 nm, and 500 nm. We observed that the 500 nm DMPs concentrated very quickly (within ∼20 s) at the bottom of the wells; however, they were concentrated within a very small area at the center of the well, limiting their ability to bind to the cAb along the periphery of the well. For this reason, the 500 nm DMPs produced very low absorbance values. The absorbance values generated by the 100 nm DMPs were significantly lower than those generated using the 200 nm DMPs, which we attribute to the reduced magnetic force experienced by the smaller particle, thus requiring a much longer time for adequate magnetic concentration (Fig. S2B). We also briefly investigated the optimal condition for sample-DMP incubation and found that incubation with agitation at 300 rpm resulted in the largest SBR and smallest variability in the absorbance values compared with faster or slower agitation speeds, or no agitation (Fig. S2C).

### 3.3 Evaluation of the magneto-ELISA performance

**Fig. 4:**
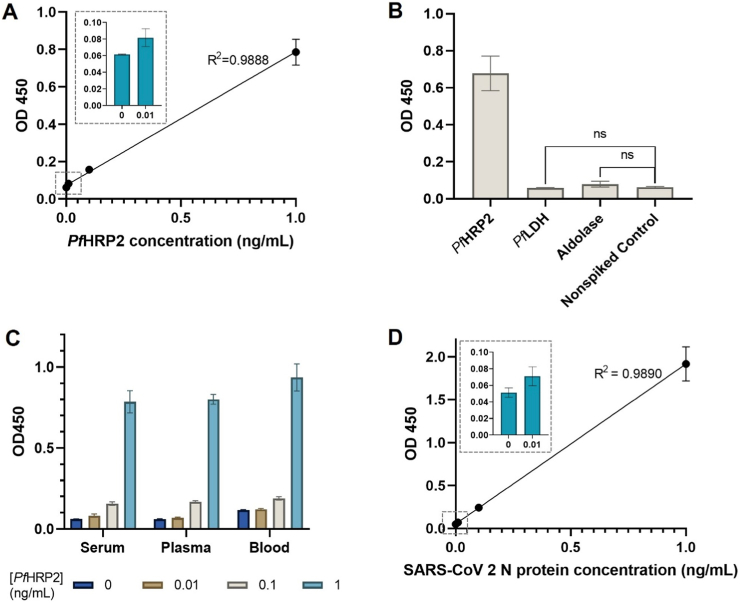
**Analytical performance of the magneto-ELISA.** (A) Standard curve generated from absorbance values measured at varying *Pf*HRP2 concentrations from 0 to 1 ng mL^−1^ in human serum. Inset shows absorbance values at 0 and 0.01 ng mL^−1^ of *Pf*HRP2. (B) Absorbance values generated from human serum spiked with 1 ng mL^−1^ of *Pf*HRP2, *Plasmodium* aldolase, *Pf*LDH, or nonspiked sera. (C) Absorbance values at varying *Pf*HRP2 concentrations from 0 to 1 ng mL^−1^ in diluted serum, plasma, or blood samples. (D) Standard curve generated from absorbance values measured at varying SARS-CoV-2 N protein concentrations from 0 to 1 ng mL^−1^ in human serum. Inset shows absorbance values at 0 and 0.01 ng mL^−1^ of SARS-CoV-2 N protein. Each data point and bar represents the mean ± SD of at least three measurements.

We first assessed the analytical performance of the magneto-ELISA by performing measurements of 10× diluted human serum spiked with increasing concentration of *Pf*HRP2 from 0 to 1 ng mL^−1^. The standard curve, generated from absorbance values at different *Pf*HRP2 concentrations, is shown in Fig. 4A, which reveals that this assay exhibits a highly linear response (*R*^*2*^ = 0.9888) from 0 to 1 ng mL^−1^. The lower LOD of this assay (calculated as 3 × standard deviation of the background signal divided by the slope of the linear regression of the standard curve [18]) is 2 pg mL^−1^ (33 fM), which is similar to the most sensitive ELISA kits that are commercially available [19,20]. The specificity of this assay was evaluated by performing measurements of human serum samples spiked with 1 ng mL^−1^ of *Pf*HRP2, *Pf*LDH or *Plasmodium* aldolase, and nonspiked serum. As shown in Fig. 4B, samples containing *Pf*LDH and aldolase resulted in very low absorbance values (∼0.065) and were similar to those generated by nonspiked serum, which was used as the blank control. In contrast, the absorbance values for the sample containing *Pf*HRP2 were 10-fold larger, indicating that this assay is highly specific to *Pf*HRP2 and will not cross-react with other *Plasmodium* proteins.

We also investigated the capability of this assay to detect protein biomarkers in other types of biofluids by performing measurements of *Pf*HRP2 spiked in 10× diluted plasma or 10× diluted whole blood. As shown in Fig. 4C, the absorbance values produced in plasma and whole blood were similar to those generated in serum with LODs of 10.7 and 12.3 pg mL^−1^, respectively. The slightly lower analytical sensitivities obtained in plasma and whole blood compared with serum is likely due to the greater background signal produced by nonspecific binding of the additional molecules and proteins in plasma and blood. These results indicate that this magneto-ELISA can be used for highly sensitive measurements using both purified and whole blood samples.

Lastly, we investigated the capability of this assay to detect other protein biomarkers, for instance, the SARS-CoV-2 N protein, by replacing the anti-*Pf*HRP2 antibodies with anti-SARS-CoV-2 N protein antibodies using the previously optimized parameters. As shown in Fig. 4D, a highly linear (*R*^*2*^ = 0.9890) response is also obtained for measurements of the SARS-CoV-2 N protein in human serum with a lower LOD of 8 pg mL^−1^ (174 fM). Based on these results, we expect that this magneto-ELISA can be readily adapted for rapid, ultrasensitive measurements of a broad range of clinically relevant protein biomarkers.

### 3.4 Validation of the magneto-ELISA using clinical blood samples

**Fig. 5:**
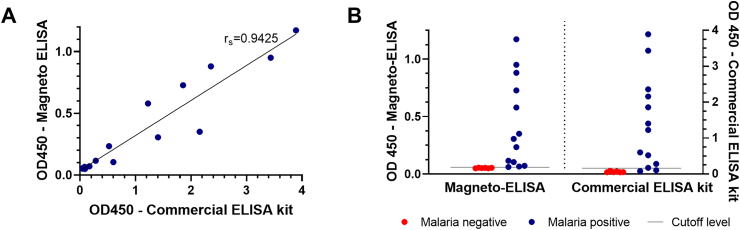
**Validation of the magneto-ELISA.** (A) Absorbance values generated by the magneto-ELISA and a commercial *Pf*HRP2 ELISA kit when detecting *Pf*HRP2 in malaria-positive (n = 13) and malaria-negative (n = 6) whole blood samples. Each point represents the mean of two (malaria-positive) or three (malaria-negative) measurements. (B) Absorbance values generated by the magneto-ELISA and the commercial ELISA kit for malaria-positive (n = 13) and malaria-negative (n = 6) samples. The horizontal lines represent the cut-off values for discriminating between positive and negative cases.

The accuracy of the magneto-ELISA was evaluated by performing measurements of blood samples from individuals with microscopy-confirmed *P. falciparum* infection and individuals with a negative malaria RDT result. *Pf*HRP2 measurements were performed on paired samples using the magneto-ELISA and a commercial Quantimal Ultrasensitive *Pf*HRP2 ELISA kit. As shown in Fig. 5A, absorbance values generated by the magneto-ELISA and commercial kit were highly correlated (Spearman's rank coefficient of 0.9425, p < 0.0001), indicating that the analytical sensitivity of the magneto-ELISA is comparable to that of the commercial kit. Fig. 5B shows absorbance values generated by the magneto-ELISA plotted side-by-side with the absorbance values generated by the Cellabs ELISA kit for all 19 clinical samples with cut-off values to compare the diagnostic accuracy of both methods. The absorbance values generated by all six negative samples were below the cut-off values for both assays, indicating that both methods were able to accurately identify all the negative samples. The Cellabs ELISA kit accurately identified 11 of the 13 positive samples based on the cut-off value specified by the manufacturer. In contrast, the absorbance values generated by all 13 positive samples were above the cut-off value of the magneto-ELISA, indicating that it was able to accurately identify all the positive samples. Therefore, while offering a superior diagnostic accuracy to the commercial ELISA kit, this magneto-ELISA is 4× faster (30 min vs. 2 hr incubation time), requires only one washing step, and does not require incubation at 37 °C, making it simpler to perform.

## 4 Conclusions

We have developed a rapid magneto-ELISA for ultrasensitive measurements of protein biomarkers in clinical specimens. This was achieved by utilizing DMPs and a simple immunomagnetic enrichment technique, which accelerates the transport of antigen-DMP conjugates to the cAb-immobilized surface, resulting in enhanced signal amplification. The analytical performance of this assay was evaluated by performing measurements of human serum samples spiked with *Pf*HRP2 or SARS-CoV-2 N protein, which exhibited LODs of 2 pg mL^−1^ and 8 pg mL^−1^, respectively. In addition to its capability to detect different types of protein biomarkers, measurements of *Pf*HRP2 spiked in human plasma, sera and whole blood demonstrate that this assay is capable of highly sensitive measurements using both purified and whole blood samples. Measurements of *Pf*HRP2 in clinical blood specimens from malaria-positive and malaria-negative individuals reveal that this magneto-ELISA offers a superior diagnostic accuracy compared to a commercial ELISA kit while being 4× faster and simpler to perform. Furthermore, this assay requires no specialized instrumentation and is compatible with standard microplate readers and ELISA protocols, allowing it to integrate readily into current clinical practice for on-site diagnostic testing and blood screening. While our current magneto-ELISA offers similar analytical performance as a commercial Quantimal Ultrasensitive *Pf*HRP2 ELISA kit, additional assay optimization could increase its sensitivity for detecting lower protein concentrations, which is associated with asymptomatic and early phases of infection. The magneto-ELISA platform can also be tailored for biomarker detection in other types of biofluids, including urine, saliva and sweat. Lastly, we envision that this technology can be readily adapted to detect other clinically relevant protein and non-protein (e.g., DNA, RNA) biomarkers by replacing the capture and detection antibodies with different bioreceptors, thereby expanding its utility for disease diagnosis and screening.

## Funding sources

This research was funded by the 10.13039/100010269Wellcome Trust [215826/Z/19/Z]. For the purpose of open access, the author has applied a CC BY public copyright license to any author accepted manuscript version arising from this submission.

## CRediT authorship contribution statement

**Kavya L. Singampalli:** Methodology, Validation, Formal analysis, Investigation, Writing – original draft, Visualization. **Jiran Li:** Conceptualization, Methodology, Validation, Writing – review & editing. **Peter B. Lillehoj:** Conceptualization, Writing – review & editing, Supervision, Funding acquisition.

## Declaration of competing interest

The authors declare that they have no known competing financial interests or personal relationships that could have appeared to influence the work reported in this paper.

## Data Availability

Data will be made available on request.

## Abbreviations

cAb: capture antibody
dAb: detection antibody
DMP: dually labeled magnetic nanoparticles
ELISA: enzyme-linked immunosorbent assay
HRP: horseradish peroxidase
LOD: limit of detection
*Pf*HRP2: *Plasmodium falciparum* histidine-rich protein 2
RDT: rapid diagnostic test
SBR: signal-to-background ratio

## Appendix A Supplementary data

Multimedia component 1 Design of the magnetic stage, estimated cost of the magnetic stage, and optimization of magneto-ELISA assay parameters. Multimedia component 1
