# Supplementary material for: Rapid magneto-enzyme-linked immunosorbent assay for ultrasensitive protein detection
Source: Anal Chim Acta. 2022 Sep 8;1225:340246. doi: 10.1016/j.aca.2022.340246 (PMC9458869; doi:10.1016/j.aca.2022.340246)
Supplement: Multimedia component 1 — Design of the magnetic stage, estimated cost of the magnetic stage, and optimization of magneto-ELISA assay parameters. [file mmc1.docx]

**Supporting Information**

**Rapid magneto-enzyme-linked immunosorbent assay for ultrasensitive protein detection**

Kavya L. Singampalli ^a,c^, Jiran Li ^b^, Peter B. Lillehoj ^a,b *^

^a^ Department of Bioengineering, Rice University, 6500 Main St. Houston, TX, 77030

^b^ Department of Mechanical Engineering, Rice University, 6100 Main St. Houston, TX, 77005

^c^ Medical Scientist Training Program, Baylor College of Medicine, One Baylor Plaza Houston, TX, 77030

Corresponding Author: Peter B. Lillehoj, [lillehoj@rice.edu](mailto:lillehoj@rice.edu), (713) 348 - 7344


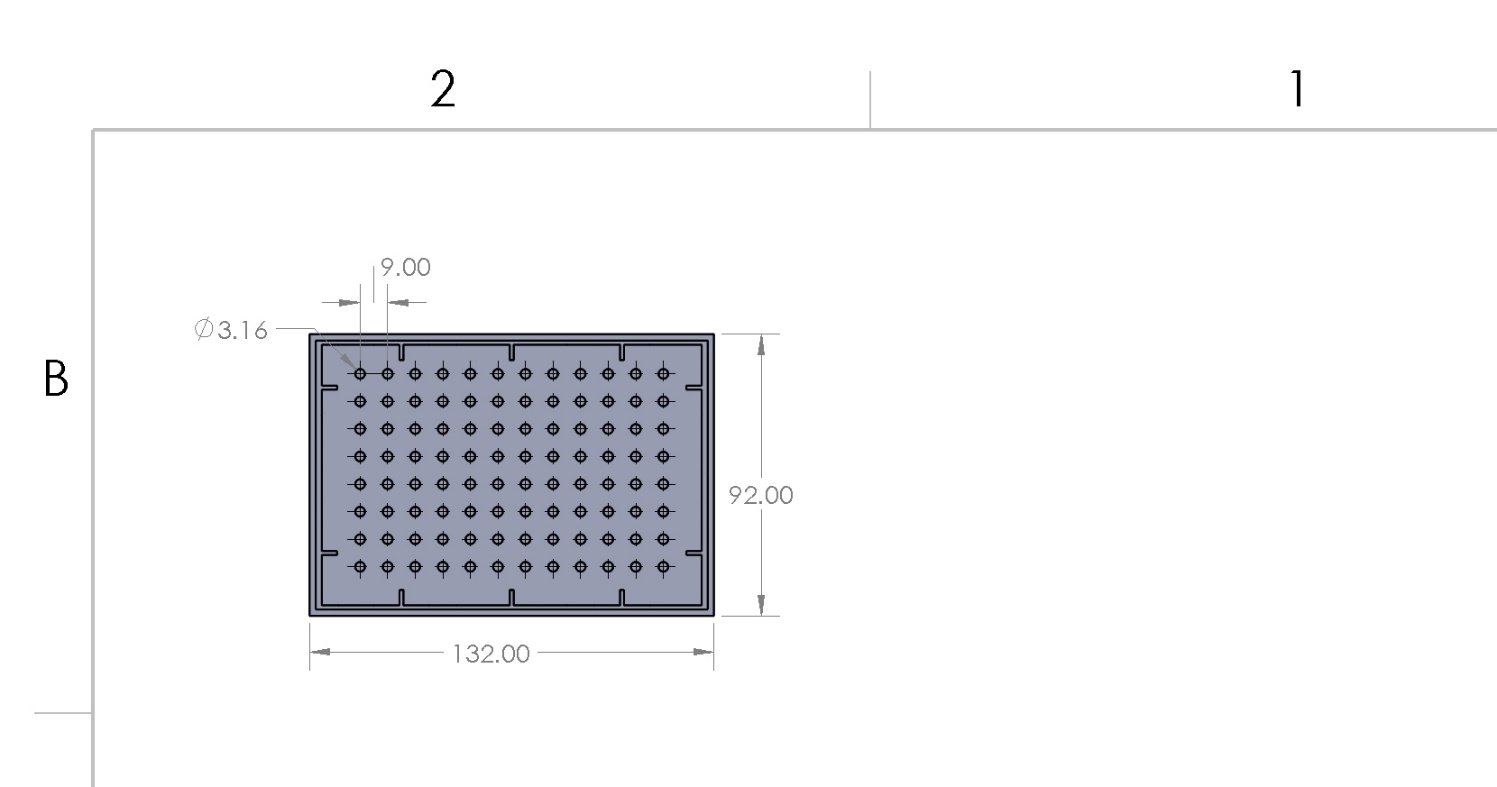


**B**

**A**


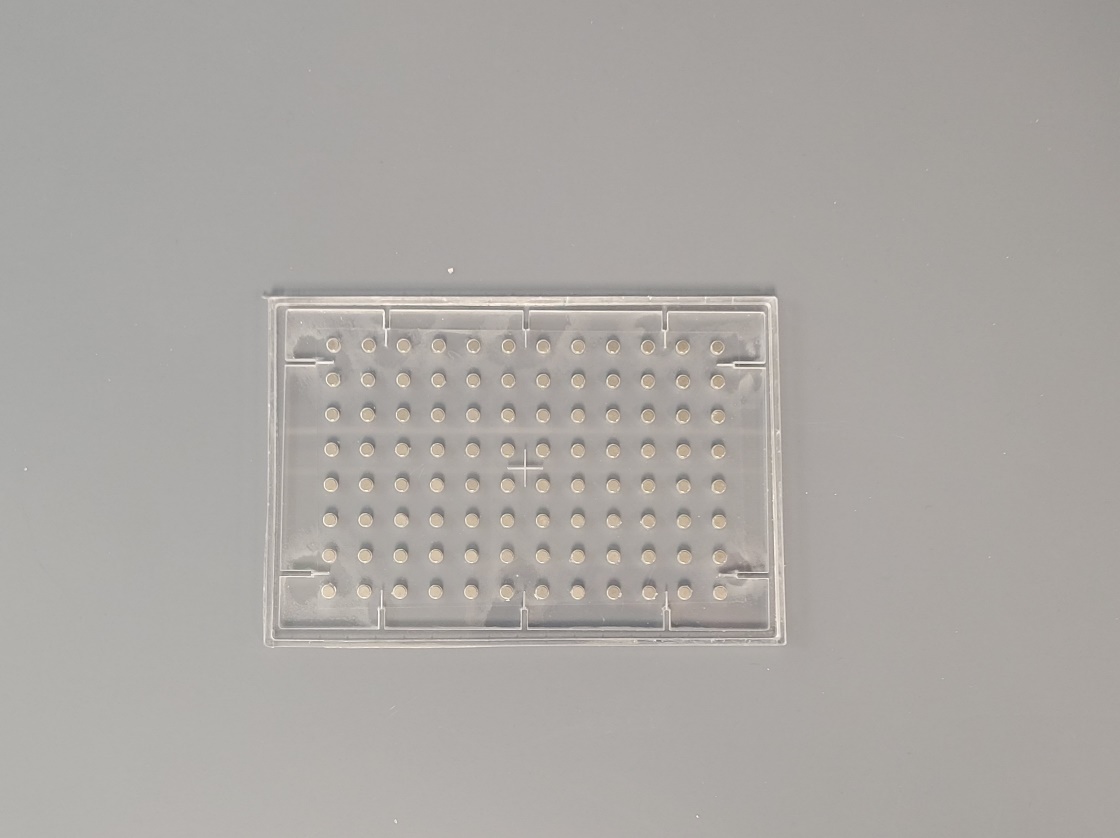


**Figure S1. Design of the magnetic stage.** (A) Dimensions of the PMMA base for the magnet array. All units are in mm. (B) Photograph of assembled magnet stage.

| **Material Costs** | **Unit Cost** | **Amount Used** | **Cost** |
| --- | --- | --- | --- |
| PMMA | $0.01 / in^2^ | 56.46 in^2^ | $0.40 |
| Double-Sided Tape | $0.01 / in^2^ | 37.64 in^2^ | $0.43 |
| Neodymium Magnets | $0.07 | 96 | $6.48 |
| **Labor** | **Unit cost** | **Time used** | **Cost** |
|  | $17.64 / hr | 0.083 hr | $1.46 |
| **Total** | | | **$8.77** |

**Table S1. Estimated cost of manufacturing the magnet stage.** Labor cost estimated from the National Industry Specific Occupational Employment and Wage Estimates.

2021. NAICS 339100 - Medical Equipment and Supplies Manufacturing. May 2021 National Industry-Specific Occupational Employment and Wage Estimates. U.S. Bureau of Labor Statistics.


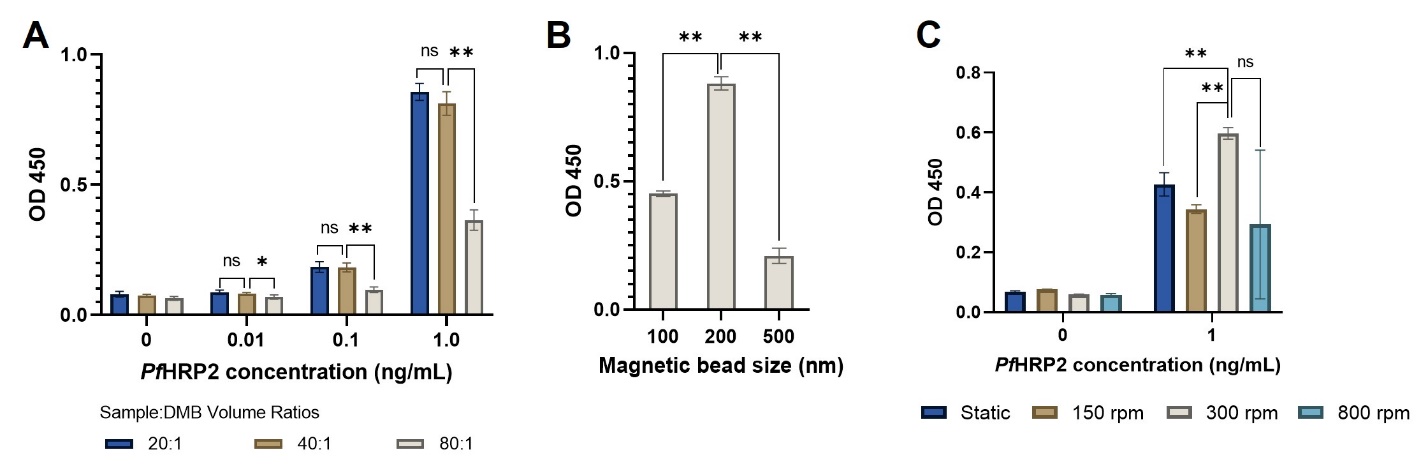


**Figure S2. Optimization of magneto-ELISA parameters.** (A) Absorbance values generated from *Pf*HRP2-spiked human sera using different sample-to-DMP volume ratios. (B) Absorbance values generated from *Pf*HRP2-spiked human sera (1 ng/mL) using varying sized magnetic particles. (C) Absorbance values generated from human sera spiked with 1 ng/mL or 0 ng/mL of *Pf*HRP2 using static or agitated incubation, at varying speeds, at room temperature before magnetic concentration. All measurements were performed with 14 min of sample-DMP incubation, 1 min of magnet concentration, and 5 min of post-magnetic concentration incubation. Each bar represents the mean ± SD of four measurements. * indicates p<0.05, ** indicates p<0.01.
